# 

**Published:** 1859-07

**Authors:** 


### Contents of the Chicago Medical Journal, July, 1859.

### ORIGINAL COMMUNICATIONS.

PAGE.

ART. I. Case of Congenital Absence of the Vagina, with the Uterus in a Rudimentary State, in which an Operation was performed; with reflections on the Operations performed for Absence or Obliteration of the Vagina. By Daniel Brainard, M.D. Prof. of Surgery in Rush Medical College........ 389

ART. II. Stomatitis Materni. By David Prince, M.D., Jacksonville, Ill........ 400

ART III. Successful Case of Resection of the Knee Joint. By J. W. Freer, M.D., Prof. of Anatomy in Rush Medical College, one of the Surgeons to Mercy Hospital, etc........ 402

### EXTRACTS.

On the Physiological Position of Fibrin. By Levin S. Joynes, M.D., Prof. of Institutes of Medicine in the Medical College of Virginia........ 405

American Medical Association........ 407

Scarlet Fever. From an Essay, by H. A. Carrington, M.D., Hyde Park, Duchess Co., N. Y........ 410

Medical Education. By Prof. Joseph P. Logan........ 423

Markoe on Subcutaneous Perforation of Bone in Ununited Fracture........ 424

On an imperfectly known function of the Pancreas, namely Digestion of Nitrogenous Food, with comparative experiments on Gastric and Intestinal Digestion, followed by a few Clinical Deductions. By L. Corvisart, M.D., in *London Lancet*........ 425

### BOOK AND PAMPHLET NOTICES.

A Manual of Elementary Chemistry, Theoretical and Practical. By George Fownes, F.R.S. From the seventh revised and corrected London edition. Edited by Robert Bridges, M.D., Professor of Chemistry in the Philadelphia College of Pharmacy, etc. Philadelphia: Blanchard & Lea. 1859. From W. B. Keen........ 433

Woman: her Diseases and Remedies. A Series of Letters to his Class. By Charles D. Meigs, M.D., Prof. of Midwifery and the Diseases of Women and Children, in the Jefferson Medical College at Philadelpeia, etc. Fourth edition, revised and enlarged. Philadelphia: Blanchard & Lea. 1859. From W. B. Keen........ 433

Importance of the Study of Legal Medicine; a Lecture introductory to the Course on Medical Jurisprudence, at the New York Medical College. By James Wynne, M. D........ 434

### MISCELLANEOUS.

Proceedings of the Semi-Annual Meeting of the Esculapian Society, held at Grand View, Ill., 25th and 26th May, 1859........ 435

Illinois State Medical Society. Annual Meeting at Decatur. Report of Proceedings. 438

Tenth Annual Session of the Indiana State Medical Society........ 442

Suspension of a Clinical School........ 448

New Colleges........ 448

Appointment and Resignation of Medical Professors........ 448

New York Colleges........ 448

Medical Journals........ 449

### EDITORIAL.

Rush Medical College........ 449

Correction........ 450

Cure of Aneurism by Compression........ 450

On Referring Unread Papers........ 451

City Hospital........ 452

